# Short- and long-term outcomes of robot-assisted versus laparoscopic lateral lymph node dissection for rectal cancer

**DOI:** 10.1007/s00423-025-03747-z

**Published:** 2025-06-07

**Authors:** Daichi Kitaguchi, Tsuyoshi Enomoto, Kinji Furuya, Shuntaro Tsukamoto, Tatsuya Oda

**Affiliations:** Department of Gastrointestinal and Hepato-Biliary-Pancreatic Surgery, Institute of Medicine, University of Tsukuba, 1-1-1, Tennnodai, Tsukuba, Ibaraki 305-8575 Japan

**Keywords:** Lymph node excision, Minimally invasive surgical procedures, Rectal neoplasm, Robotic surgical procedures, Urinary retention

## Abstract

**Purpose:**

The lateral pelvic cavity is an anatomically tricky site to access using a linear approach; therefore, robot-assisted lateral lymph node dissection (LLND) may be superior over existing laparoscopic procedures. In this study, we aimed to compare the short- and long-term outcomes of robot-assisted LLND (R-LLND) versus laparoscopic LLND (L-LLND) for locally advanced low rectal cancer and explore the potential advantages of robot-assisted surgery.

**Methods:**

This single-center, retrospective cohort study included patients aged ≥ 18 years who underwent minimally invasive total mesorectal excision (TME) plus LLND for low rectal adenocarcinoma. Patients were divided into L-LLND and R-LLND groups. The short- and long-term outcomes of the procedures were compared.

**Results:**

There were 41 patients in the L-LLND group and 21 in the R-LLND group. The incidence of postoperative complications was significantly lower in the R-LLND group (49% vs. 19%, *p* = 0.029), especially urinary retention (29% vs. 5%, *p* = 0.046). The median postoperative hospital stay was significantly shorter in the R-LLND group (22 vs. 15 days, *p* < 0.001). The 3-year relapse-free survival rates in the L-LLND and R-LLND groups were 75.3% (95% confidence interval [CI]: 58.9–85.9) and 65.7% (95% CI: 30.7–86.1), respectively. No significant differences were observed in long-term survival outcomes.

**Conclusion:**

Patients with locally advanced rectal cancer who underwent TME plus R-LLND had a significantly lower incidence of postoperative complications and a significantly shorter postoperative hospital stay compared to those who underwent TME plus L-LLND. The long-term outcomes were comparable, and no oncological concerns associated with R-LLND were observed.

## Introduction

Total mesorectal excision (TME), which is the international standard surgical procedure for lower rectal cancer, involves the removal of the rectum and all pararectal lymph nodes within an oncologic package [1]. However, metastases to lateral pelvic lymph nodes (LLN) develop occasionally, and these nodes are located outside the surgical field of TME [2, 3]. A retrospective multicenter study in Japan reported that the incidence of LLN metastasis (LLNM) in patients with T3 or T4 lower rectal cancer was 18.1% [2].

LLN dissection (LLND) is recommended in the Japanese guideline if the tumor is located below the peritoneal reflection to reduce the risk of local recurrence [4]. Contrarily, in Western countries, neoadjuvant chemoradiotherapy (CRT) or total neoadjuvant therapy followed by TME is the standard treatment for clinical stage II–III rectal cancer [5–9]. LLND is rarely performed unless lateral node involvement is suspected based on imaging, particularly when enlarged lateral nodes persist after CRT [10, 11]. Although the overall local recurrence rate with this strategy is 5–9% [7, 12, 13], studies have demonstrated that patients with lateral nodal enlargement have a higher rate of local recurrence of up to 30%, even after CRT plus TME. The addition of LLND results in a significantly lower lateral local recurrence rate [14, 15].

Robot-assisted LLND (R-LLND) was first described by Park et al., who reported its safety and feasibility in a series of eight patients [16]. The advantages of robot-assisted surgery, such as multi-joint forceps with motion scaling, a high-quality three-dimensional camera, and significantly improved ergonomics, are all ideal for LLND, a complex procedure. In addition, the lateral pelvic cavity is an anatomically tricky site to access using a linear approach; therefore, R-LLND may be over existing laparoscopic procedures. Recent systematic reviews and meta-analyses have concluded that R-LLND offers advantages over laparoscopic LLND (L-LLND), such as lower intraoperative blood loss, more LLN harvest, fewer postoperative complications, and a shorter postoperative hospital stay [17, 18]. However, there have been limited reports on this topic, and the evidence remains insufficient, particularly regarding long-term outcomes.

In this study, we aimed to compare the short- and long-term outcomes of R-LLND versus L-LLND for locally advanced low rectal cancer and explore the potential advantages of robot-assisted surgery.

## Materials and methods

### Study design

This single-center, retrospective cohort study used prospectively collected clinical data. The study protocol was approved by the ethics committee of the University of Tsukuba Hospital (Approval No. R01-104) and conformed to the 1964 Declaration of Helsinki (revised in Brazil in 2013). This study adhered to the Strengthening the Reporting of Observational Studies in Epidemiology guidelines [19]. Informed consent was obtained from the patients on an opt-out basis.

## Participants

Patients aged ≥ 18 years who underwent minimally invasive TME plus LLND for primary and solitary low rectal adenocarcinoma with clinical T3-4 and/or clinical LLNM between January 2017 and March 2024 were included in this study. Low rectal cancer was defined as a tumor with a lower margin located below the second valve of Houston. Both therapeutic and prophylactic LLND were included. Patients who did not undergo neoadjuvant CRT were indicated for bilateral prophylactic LLND. Contrarily, for patients who underwent neoadjuvant CRT, therapeutic LLND was performed only for the side with clinically positive LLNM. Patients with distant metastasis were excluded, and paraaortic lymph nodes were defined as distant metastasis. The other exclusion criteria included recurrent tumors, multiple primary tumors, and non-adenocarcinomas, such as neuroendocrine and gastrointestinal stromal tumors. Patients who underwent open surgery, total pelvic exenteration, or emergency surgery were also excluded. Routine preoperative assessments were performed using digital rectal examination; colonoscopy; computed tomography of the chest, abdomen, and pelvis; and magnetic resonance imaging (MRI) of the pelvis. All patients participated in multidisciplinary team discussions. Treatment strategies, including neoadjuvant and adjuvant therapies, were determined based on the clinical treatment protocols of the University of Tsukuba Hospital, following the rectal cancer clinical practice guidelines of the European Society for Medical Oncology [10] and Japanese Society for Cancer of the Colon and Rectum [4]. A threatened circumferential resection margin (CRM) and cN2 were the primary indications for neoadjuvant chemoradiation therapy (CRT). The CRT regimen consisted of long-course whole pelvic irradiation (a total of 50 Gy in 25 fractions) combined with concomitant capecitabine (825 mg/m² of body surface area, administered twice daily on the days of radiotherapy). The usual interval between completion of CRT and surgery was 6–11 weeks. Adjuvant chemotherapy was administered to microsatellite stable, high-risk stage II patients (including those with pT4 stage, perforation, poorly differentiated tumors, lymphovascular invasion, and perineural invasion) and to stage III patients, if tolerable. The primary regimen for adjuvant chemotherapy was capecitabine plus oxaliplatin, although capecitabine alone was considered for patients who were not fit for oxaliplatin administration.

## Surgical procedure

All procedures were performed by experienced surgeons qualified in the Endoscopic Surgical Skill Qualification System established by the Japan Society for Endoscopic Surgery [20]. Until December 2018, all procedures were performed laparoscopically; however, since January 2019, both laparoscopic and robot-assisted procedures have been performed concurrently.

The dissected area for LLND, encompassing the internal iliac and obturator lymph node regions, was substantially consistent between prophylactic and therapeutic LLND. This area did not differ between laparoscopic and robot-assisted procedures. The obturator lymph node region is located between the vesico-hypogastric fascia medially and the internal obturator muscle laterally. The internal iliac lymph node region lies within the vesico-hypogastric fascia, which forms the medial border of the obturator lymph node area. The internal iliac region is located along the internal iliac artery and is bordered medially by the uretero-hypogastric fascia and laterally by the obturator lymph node region [21].

The surgical procedure for LLND has been described previously [21–24] and was similar in both the laparoscopic and robot-assisted methods, except for the trocar position. LLND was performed following TME and transection of the distal rectum. The obturator nerves were always preserved unless the metastatic node invaded it, while the obturator artery and vein were usually resected.

## Outcomes

Patient characteristics included age, sex, body mass index, American Society of Anesthesiologists physical status, carcinoembryonic antigen, carbohydrate antigen 19 − 9, neoadjuvant CRT, and adjuvant chemotherapy. Preoperative tumor characteristics assessed clinically or radiologically included tumor size, tumor height from the anal verge, tumor differentiation, tumor depth, clinical lymph node status, clinical LLNM, involvement of the mesorectal fascia, and extramural venous invasion (EMVI). Lymph node metastasis, involvement of the mesorectal fascia, and EMVI were specifically assessed based on MRI findings. Lymph node metastasis was defined as a short diameter ≥ 7 mm, and EMVI was defined as scores of 3 and 4 [25]. The surgical characteristics included the type of procedure, anal preservation, diverting stoma, and sidedness of the LLND. The histopathological outcomes included the stage, pathological LLNM, margins, and American Joint Committee on Cancer tumor regression grade. Pathological CRM positivity was defined as the presence of tumor cells within 1 mm of the CRM.

The postoperative outcomes included the operation time, volume of blood loss, open conversion, number of harvested LLNs, complications, reoperation, and length of hospital stay. In addition to the total operation time, the time required for LLND was also evaluated. Postoperative complications of Clavien–Dindo classification grade II and above were included in the analysis. Urinary retention was defined as requiring urinary self-catheterization or catheter placement, in addition to the need for pharmacological intervention. The oncological outcomes included local recurrence, distant recurrence, relapse-free survival (RFS), overall survival (OS), and local recurrence-free survival (LRFS).

### Statistical analysis

All patients were divided into two groups for comparative analysis: L-LLND and R-LLND. Continuous variables are presented as medians (ranges) and were analyzed using the Mann–Whitney U test. Categorical variables are presented as frequencies (%) and were analyzed using Fisher’s exact test. Survival outcomes are presented as rates (%) with 95% confidence intervals (CIs). Survival curves were plotted using the Kaplan–Meier method. The RFS and OS were compared using log-rank tests. All p-values were two-sided, and *p* ≤ 0.05 was considered statistically significant. All statistical analyses were conducted using EZR (Saitama Medical Center, Jichi Medical University, version 1.68) [26], a graphical user interface for R (R Foundation for Statistical Computing, Vienna, Austria, version 4.3.1).

## Results

### Baseline characteristics

Over the 87-month study period, 62 patients met the inclusion criteria, including 41 in the L-LLND group and 21 in the R-LLND group. The patient, tumor, surgical, and histopathological characteristics are presented in Table 1. Overall, approximately 53% of the patients had clinical LLNM and underwent therapeutic LLND. The remaining 47% underwent prophylactic LLND. Although there were differences in the details of the type of procedure, there were no significant differences in other items between the two groups.

**Table 1:** Baseline patient, tumor, surgical, and histopathological characteristics

	ALL N=62	L-LLND N=41	R-LLND N=21	*P-value*
Age (years)*	63 [41–78]	63 [41–77]	63 [46–78]	*0.592*
Sex (Male)	44 (71%)	30 (73%)	14 (67%)	*0.768*
BMI (kg/m^2^)*	23.4 [16.8–32.7]	23.7 [16.8–30.3]	22.5 [20.0–32.7]	*0.169*
ASA-PS				*0.898*
1	5 (8%)	3 (7%)	2 (10%)	
2	49 (79%)	32 (78%)	17 (81%)	
3	8 (13%)	6 (15%)	2 (10%)	
CEA (ng/mL)*	4.3 [0.3–74]	4.1 [0.5–74]	4.9 [0.3–54.4]	*0.401*
CA19-9 (U/mL)*	11.2 [0.6–164]	10.7 [0.6–164]	11.7 [0.6–57.8]	*0.806*
Tumor size (mm)*	45 [19–100]	45 [19–100]	42 [30–80]	*0.982*
Tumor height from AV (mm)*	54 [0–100]	55 [0–90]	53 [0–100]	*0.954*
Tumor differentiation				*0.830*
well	13 (21%)	8 (20%)	5 (24%)	
moderate	43 (69%)	28 (68%)	15 (71%)	
poor/mucinous	6 (10%)	5 (12%)	1 (5%)	
Tumor depth				*1.000*
cT2	1 (2%)	1 (2%)	0	
cT3	47 (76%)	31 (76%)	16 (76%)	
cT4	14 (23%)	9 (22%)	5 (24%)	
LN status				*0.944*
cN0	18 (29%)	11 (27%)	7 (33%)	
cN1	6 (10%)	4 (10%)	2 (10%)	
cN2	5 (8%)	4 (10%)	1 (5%)	
cLLNM	33 (53%)	22 (54%)	11 (52%)	
MRF involvement	34 (55%)	20 (49%)	14 (67%)	*0.281*
EMVI	29 (47%)	19 (46%)	10 (48%)	*1.000*
Neoadjuvant CRT	34 (55%)	22 (54%)	12 (57%)	*1.000*
Type of procedure				*0.047*
LAR	29 (47%)	15 (37%)	14 (67%)	
ISR	13 (21%)	12 (29%)	1 (5%)	
APR	19 (31%)	13 (32%)	6 (29%)	
Hartmann	1 (2%)	1 (2%)	0	
Anal preservation	42 (68%)	27 (66%)	15 (71%)	*0.777*
Diverting stoma	42 (68%)	27 (66%)	15 (71%)	*-*
Sidedness of LLND				*0.170*
Bilateral	36 (58%)	26 (63%)	10 (48%)	
Left	14 (23%)	10 (24%)	4 (19%)	
Right	12 (19%)	5 (12%)	7 (33%)	
pT stage				*0.785*
pT0 (pCR)	3 (5%)	1 (2%)	2 (10%)	
pT1	2 (3%)	2 (5%)	0	
pT2	12 (19%)	8 (20%)	4 (19%)	
pT3	42 (68%)	28 (68%)	14 (67%)	
pT4	3 (5%)	2 (5%)	1 (5%)	
pN stage				*0.698*
pN0	38 (61%)	25 (61%)	13 (62%)	
pN1	12 (19%)	9 (22%)	3 (14%)	
pN2	12 (19%)	7 (17%)	5 (24%)	
pLLMM	8 (13%)	7 (17%)	1 (5%)	*0.247*
Number of pLLNMs*				*0.107*
Left	0 [0–5]	0 [0–5]	0	*0.440*
Right	0 [0–2]	0 [0–2]	0 [0–1]	
pDM positive	1 (2%)	1 (2%)	0	*1.000*
pCRM positive	2 (3%)	2 (5%)	0	*0.545*
AJCC TRG				*0.441*
TRG 0 (pCR)	3 (8%)	1 (5%)	2 (17%)	
TRG 1	13 (38%)	10 (45%)	3 (25%)	
TRG 2	17 (50%)	10 (45%)	7 (58%)	
TRG 3	1 (3%)	1 (5%)	0	
Adjuvant chemotherapy	36 (58%)	21 (51%)	15 (71%)	*0.176*

*Median [range]

*L-LLND* laparoscopic lateral lymph node dissection; *R-LLND* robot-assisted lateral lymph node dissection; BMI, body mass index; *ASA-PS* American Society of Anesthesiologists physical status; *CEA* carcinoembryonic antigen; *CA*19-9 carbohydrate antigen 19 − 9; *AV* anal verge; *LN* lymph node; *cLLNM* clinical lateral lymph node metastasis; *MRF* mesorectal fascia; *EMVI* extramural venous invasion; *CRT* chemoradiotherapy; *LAR* low anterior resection; ISR, intersphincteric resection; *APR* abdominoperineal resection; *pCR* pathological complete response; *pLLNM* pathological lateral lymph node metastasis; *pDM* pathological distal margin; *pCRM* pathological circumferential resection margin; *AJCC–TRG* American Joint Committee on Cancer tumor regression grade; *ACT* adjuvant chemotherapy

## Short-Term postoperative outcomes

The postoperative outcomes are presented in Table 2. The median total operation time in the L-LLND and R-LLND groups was 455 and 549 min, respectively, with a significantly longer time in the R-LLND group (*p* = 0.017). However, the time required for LLND was comparable between the two groups on both sides. There was no open conversion in both groups. The incidence of postoperative complications in the L-LLND and R-LLND groups was 49% and 19%, respectively, with significantly lower incidence in the R-LLND group (*p* = 0.029). Particularly, there was a significant difference in the incidence of urinary retention (29% vs. 5%, *p* = 0.046). The median postoperative hospital length of stay in the L-LLND and R-LLND groups was 22 and 15 days, respectively, with a significantly shorter length of stay in the R-LLND group (*p* < 0.001). The number of harvested LLNs on the right side in the R-LLND group was significantly lower; however, the incidence of recurrence was not significantly different. LLN recurrence occurred in two cases in the L-LLND group; however, the recurrence sites were outside the areas initially dissected during LLND. No LLN recurrence was observed in the R-LLND group.

**Table 2:** Postoperative outcomes

	**ALL** **N=62**	**L-LLND** **N=41**	**R-LLND** **N=21**	***P-value***
Operation time (min)*				
Total	471 [281–640]	455 [281–640]	549 [311–638]	*0.017*
Left LLND	68 [27–119]	66 [27–119]	69 [43–102]	*0.618*
Right LLND	60 [24–110]	61 [32–110]	57 [24–94]	*0.343*
Blood loss (mL)*	120 [0–1200]	130 [0–1200]	70 [0–660]	*0.153*
Open conversion	0	0	0	*-*
Number of harvested LLN*				
Left	6 [0–16]	6 [0–16]	6 [0–16]	*0.920*
Right	6 [0–16]	6 [0–16]	3 [0–8]	*0.008*
Postoperative complications (CD≥2)				
Total	24 (39%)	20 (49%)	4 (19%)	*0.029*
Urinary retention	13 (21%)	12 (29%)	1 (5%)	*0.045*
Urinary tract infection	8	7	1	
Pelvic abscess	6	5	1	
Reoperation	1 (2%)	1 (2%)	0	*1.000*
Postoperative hospital stay (days)*	20 [9–60]	22 [10–60]	15 [9–22]	*<0.001*
Recurrence				*0.551*
Total	15 (24%)	11 (27%)	4 (19%)	
Local	10 (16%)	6 (15%)	4 (19%)	
LLN	2	2	0	
Distant	12 (19%)	10 (24%)	2 (10%)	
Lung	8	7	1	
Liver	2	2	0	
PALN	2	1	1	
Peritoneum	1	1	0	
Both	7 (11%)	5 (12%)	2 (10%)	

*Median [range]

*L-LLND* laparoscopic lateral lymph node dissection; *R-LLND* robot-assisted lateral lymph node dissection; *CD* Clavien–Dindo classification grade; *LLN* lateral lymph node; *PALN* paraaortic lymph node

### Long-Term survival outcomes

**Fig. 1:**
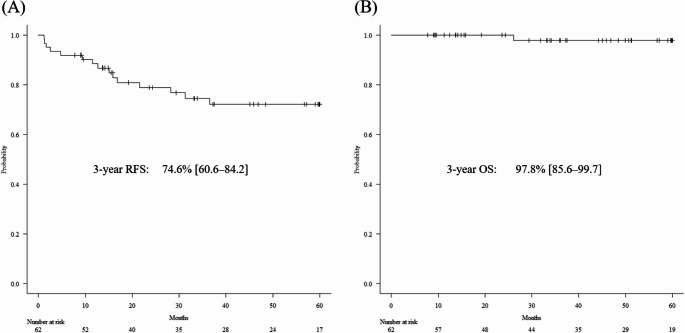
Long-term survival outcomes for the entire cohort. (**A**) Kaplan–Meier curve for the relapse-free survival (RFS). The 3-year RFS was 74.6% (95% confidence interval [CI]: 60.6–84.2). (**B**) Kaplan–Meier curve for the overall survival (OS). The 3-year OS was 97.8% (95% CI: 85.6–99.7)

The long-term survival outcomes for the entire cohort are illustrated in Fig. 1. Over a median follow-up period of 47.0 months (range: 7.9–88.2 months), the 3-year RFS and OS rates were 74.6% (95% CI: 60.6–84.2) and 97.8% (95% CI: 85.6–99.7), respectively.

**Fig. 2:**
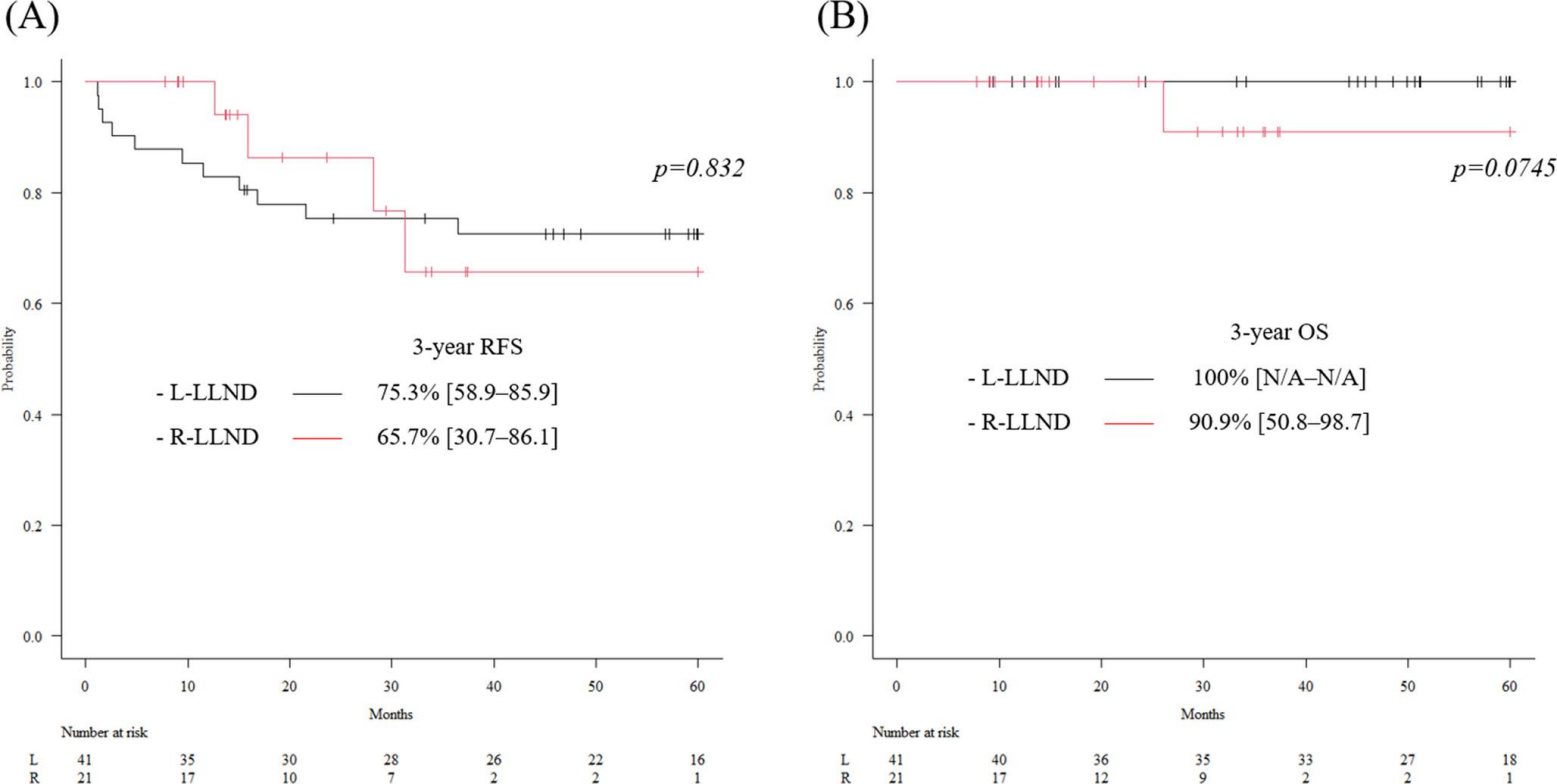
Long-term survival outcomes for each group Black: laparoscopic lateral lymph node dissection (L-LLND) group; Red: robot-assisted lateral lymph node dissection (R-LLND) group. (**A**) Kaplan–Meier curve for relapse-free survival (RFS). The 3-year RFS rates in the L-LLND and R-LLND groups were 75.3% (95% confidence interval [CI]: 58.9–85.9) and 65.7% (95% CI: 30.7–86.1), respectively, with no significant difference (*p* = 0.832). (**B**) Kaplan–Meier curve for overall survival (OS). The 3-year OS rates in the L-LLND and R-LLND groups were 100% and 90.9% (95% CI: 50.8–98.7), respectively, also without a significant difference (*p* = 0.075)

The Kaplan–Meier curves for each group are presented in Fig. 2. The 3-year RFS rates in the L-LLND and R-LLND groups were 75.3% (95% CI: 58.9–85.9) and 65.7% (95% CI: 30.7–86.1), respectively, with no significant differences in RFS between them (*p* = 0.832). Similarly, there was no significant difference in OS between the two groups (*p* = 0.0745).

**Fig. 3:**
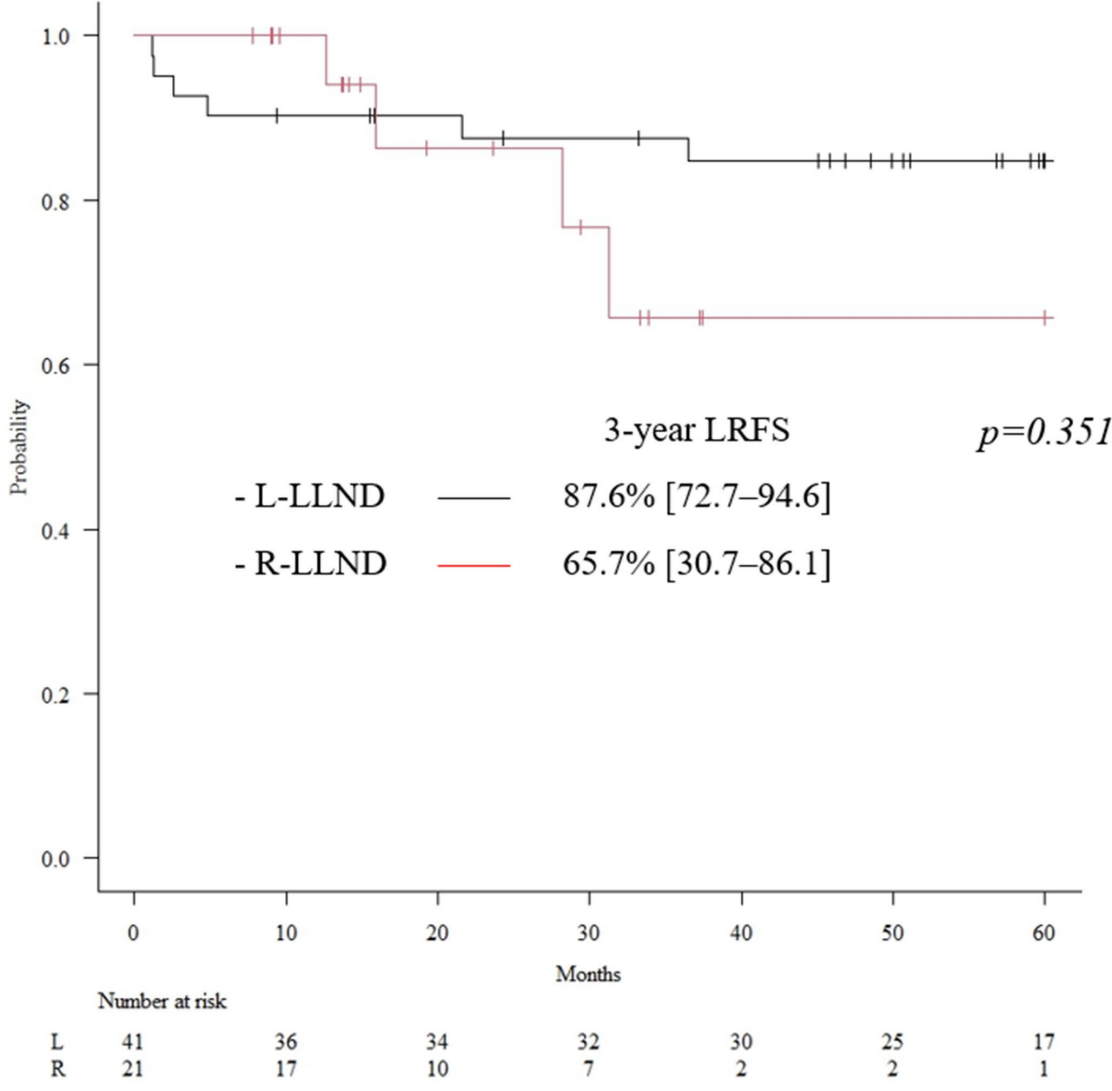
Kaplan–Meier curves for local recurrence-free survival (LRFS) in each group. The 3-year LRFS was 87.6% (95% CI: 72.7–94.6) in the L-LLND group and 65.7% (95% CI: 30.7–86.1) in the R-LLND group, with no significant difference between them (*p* = 0.351). L-LLND, laparoscopic lateral lymph node dissection; R-LLND, robot-assisted lateral lymph node dissection; CI, confidence interval

Figure 3 shows the Kaplan–Meier curves for LRFS in each group. The 3-year LRFS was 87.6% (95% CI: 72.7–94.6) in the L-LLND group and 65.7% (95% CI: 30.7–86.1) in the R-LLND group, with no significant difference between them (*p* = 0.351).

## Discussion

In this study, we compared the short- and long-term outcomes of L-LLND and R-LLND. The results demonstrated that R-LLND significantly reduced the incidence of postoperative complications, especially urinary retention, and significantly shortened the postoperative hospital stay. Although the total operation time was significantly longer in the R-LLND group, the time required for LLND itself was comparable to that of the L-LLND group. Regarding long-term outcomes, the recurrence rate, RFS, and OS were not significantly different between the two groups. These results indicated that a robot-assisted approach might be the optimal surgical approach for minimally invasive LLND, improving short-term outcomes without deteriorating long-term outcomes.

Robotic assistance has the potential to overcome some of the limitations of laparoscopic rectal cancer surgery, providing an immersive 3-dimensional depth of field, articulating instruments, and a stable camera platform. The ROLARR trial failed to demonstrate the superiority of robot-assisted laparoscopic surgery over conventional laparoscopic surgery [27]. However, the REAL trial reported that roboti-assisted surgery could significantly reduce the secondary endpoints, including CRM positivity and 30-day postoperative complications (Clavien–Dindo classification grade II or higher) compared to laparoscopic surgery [28]. Robotic assistance may be particularly advantageous in procedures in deep and narrow lateral pelvic cavities with angle limitations of instrument insertion due to complex anatomical structures.

Recently, several systematic reviews and meta-analyses comparing short-term outcomes of R-LLND versus L-LLND have shown that although R-LLND increased the total operation time, it reduced the volume of blood loss and incidence of postoperative complications (especially urinary tract-related complications), shortened the postoperative hospital stay, and increased the number of harvested LLNs [17, 18]. This study failed to demonstrate a significant reduction in the volume of blood loss and an increase in the number of harvested LLNs in the R-LLND group. However, there was a significant decrease in the incidence of postoperative complications, especially urinary retention, and a significant shortening in the postoperative hospital stay in the R-LLND group, which is consistent with previous reports. Robotic assistance enabled precise, sharp dissection while maintaining proper surgical planes, which likely contributed to the avoidance of pelvic autonomic nerve injury.

Results from a randomized controlled trial comparing mesorectal excision with and without LLND for clinical stage II or III lower rectal cancer (JCOG0212) concluded that LLND is not associated with a significant increase in the incidence of urinary dysfunction [29, 30]. However, caution should be exercised in interpreting these results. First, the JCOG0212 trial included only upfront prophylactic LLND for patients without clinical LLNM and did not include therapeutic LLND followed by CRT for patients with clinical LLNM [29]. Second, in the JCOG0212 trial, urinary dysfunction was defined as ≥ 50 mL residual urine occurring at least once, and no measurement of residual urinary volume was also defined as urinary dysfunction [30]. We believe these results may change under different diagnostic criteria for urinary dysfunction in different patient cohorts and that the negative impact of LLND on postoperative urinary function should be fully considered.

Contrarily, reports on long-term outcomes comparing these two approaches are limited [31–34]. In this study, the local recurrence rate of the entire cohort was 16%, which is relatively higher than expected. Several factors may contribute to this outcome. First, we believe our cohort included more advanced cases than the typical patient population, as 47% had EMVI and 53% had clinically positive LLNM. Second, despite these characteristics, our indication for neoadjuvant CRT during the study period was based solely on CRM-threatening and/or cN2 status, which may have been insufficient. Nevertheless, despite the relatively higher overall recurrence rate, there were no significant differences in either RFS or OS between the two groups, indicating that R-LLND did not raise any oncologic concerns. We hope this study’s results will contribute to evidence-building through future systematic reviews and meta-analyses.

This study has some limitations. First, this was a single-center retrospective study with a small sample size. Second, we introduced R-LLND in 2019. Therefore, although both laparoscopic and robot-assisted procedures have been performed concurrently since 2019, the impact of chronological bias could not be completely eliminated. Third, the follow-up period, especially in the R-LLND group, was short and insufficient for assessing long-term outcomes. Therefore, we acknowledge that the primary outcomes of this study are short-term outcomes, and long-term outcomes should be considered as supporting information. Fourth, the incidence of urinary retention was significantly reduced in the R-LLND group; however, it remains unclear whether this complication was related to TME or LLND. However, TME with LLND poses a higher risk of urinary retention compared to TME alone. Therefore, we believe that TME with LLND represents the optimal cohort for emphasizing the advantages of robot-assisted surgery.

## Conclusion

This study demonstrated that patients with locally advanced rectal cancer who underwent TME plus R-LLND had a significantly lower incidence of postoperative complications, especially urinary retention, and a significantly shorter postoperative hospital stay compared to those who underwent TME plus L-LLND. The long-term outcomes were comparable, and no oncological concerns associated with R-LLND were demonstrated. The advantages of robot-assisted surgery are particularly evident in deep and narrow surgical fields with limited angles for instrument insertion, with the lateral pelvic cavity serving as a prime example.

## Data Availability

The data that support the findings of this study are available from the corresponding author upon reasonable request.
